# Paraventricular nucleus‐central amygdala oxytocinergic projection modulates pain‐related anxiety‐like behaviors in mice

**DOI:** 10.1111/cns.14282

**Published:** 2023-05-29

**Authors:** Yu‐Jie Li, Wei‐Jia Du, Rui Liu, Gui‐Ying Zan, Bing‐Lu Ye, Qian Li, Zhi‐Hao Sheng, Ya‐Wei Yuan, Yu‐Jie Song, Jing‐Gen Liu, Zhi‐Qiang Liu

**Affiliations:** ^1^ Shanghai Key Laboratory of Maternal Fetal Medicine, Shanghai Institute of Maternal‐Fetal Medicine and Gynecologic Oncology, Department of Anesthesiology, Clinical and Translational Research Center, Shanghai First Maternity and Infant Hospital, School of Medicine Tongji University Shanghai China; ^2^ Key Laboratory of Receptor Research, Shanghai Institute of Materia Medica Chinese Academy of Sciences Shanghai China

**Keywords:** anxiety, central nucleus of the amygdala, oxytocin, pain, paraventricular nucleus

## Abstract

**Aims:**

Anxiety disorders associated with pain are a common health problem. However, the underlying mechanisms remain poorly understood. We aimed to investigate the role of paraventricular nucleus (PVN)‐central nucleus of the amygdala (CeA) oxytocinergic projections in anxiety‐like behaviors induced by inflammatory pain.

**Methods:**

After inflammatory pain induction by complete Freund's adjuvant (CFA), mice underwent elevated plus maze, light–dark transition test, and marble burying test to examine the anxiety‐like behaviors. Chemogenetic, optogenetic, and fiber photometry recordings were used to modulate and record the activity of the oxytocinergic projections of the PVN‐CeA.

**Results:**

The key results are as follows: inflammatory pain‐induced anxiety‐like behaviors in mice accompanied by decreased activity of PVN oxytocin neurons. Chemogenetic activation of PVN oxytocin neurons prevented pain‐related anxiety‐like behaviors, whereas inhibition of PVN oxytocin neurons induced anxiety‐like behaviors in naïve mice. PVN oxytocin neurons projected directly to the CeA, and microinjection of oxytocin into the CeA blocked anxiety‐like behaviors. Inflammatory pain also decreased the activity of CeA neurons, and optogenetic activation of PVN^oxytocin^‐CeA circuit prevented anxiety‐like behavior in response to inflammatory pain.

**Conclusion:**

The results of our study suggest that oxytocin has anti‐anxiety effects and provide novel insights into the role of PVN^oxytocin^‐CeA projections in the regulation of anxiety‐like behaviors induced by inflammatory pain.

## Graphical abstract

Our study suggests that oxytocin has anti‐anxiety effects and provides novel insights into the role of PVN‐CeA oxytocin projections in the regulation of anxiety‐like behaviors induced by inflammatory pain.

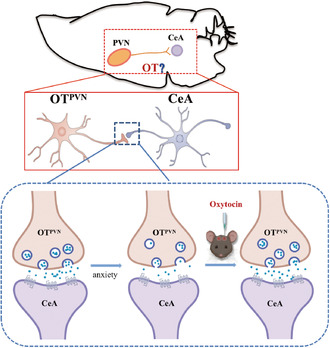

## 1 INTRODUCTION

Anxiety disorders have the highest incidence among all emotional disorders in the world, and are characterized by excessive fear, anxiety, and avoidance of stimuli. Currently, limited research is available on pathogenesis of anxiety disorders and chonic pain.^1^ However, no studies to date have assessed the association of certain brain regions and neurotransmitters with anxiety symptoms.^2^

Oxytocin is a nonapeptide produced and secreted by the paraventricular nucleus (PVN) and supraoptic nucleus (SON), which has been reported to play an important role in pain, addiction, and emotional regulation.^3^, ^4^, ^5^, ^6^ Clinical and preclinical studies have shown that oxytocin exerts analgesic effects through oxytocin receptors^7^, ^8^, ^9^, ^10^ and it has been found to improve anxiety‐like behavior in both female and male mice.^11^ Oxytocinergic system also has protective effects against negative effects on brain and behavior of animals.^12^ However, the possible functional involvement of oxytocin in anxiety and how oxytocin exerts this effect remain unclear.

The amygdala is one of the most important brain regions for emotion regulation. Many studies have shown that oxytocin neurons project to amygdala,^3^ and affect a variety of emotions and social behaviors.^13^, ^14^ Due to the important role of oxytocin in the regulation of emotions, changes in oxytocin in the amygdala most likely contribute to the incidence of anxiety symptoms. The findings of a previous study found that projections from the PVN to the central nucleus of the amygdala (CeA) are critical for discriminating emotions in rodents.^15^

In this study, we firstly explored how PVN‐CeA oxytocinergic projection modulate the anxiety‐like behavior in mice. It has significance for the treatment of anxiety. We used pharmacological and biochemical methods to investigate the role of oxytocin in anxiety‐like behaviors. Chemogenetic and fiber recordings were used to modulate and record the activity of the oxytocinergic neurons in the PVN. Subsequently, we used optogenetic manipulations to elucidate the role of PVN^oxytocin^‐CeA projections in anxiety‐like behaviors. Our results revealed an essential role of the PVN‐CeA oxytocin pathway in anxiety‐like behaviors.

## 2 METHODS

### 2.1 Materials

Anti‐oxytocin (ab212193) antibodies were purchased from Abcam. Fluor 488 goat anti‐rabbit IgG (A‐11070) and Fluor 594 goat anti‐mouse IgG (ab150116) secondary antibodies were purchased from Invitrogen. Oxytocin acetate (MB1177) was purchased from Meilunbio. L‐368,899 hydrochloride (2641/1) was purchased from Tocris.

### 2.2 Animals and inflammatory pain model

Male C57BL/6 mice weighing 20–25 g (8–10 weeks old) were purchased from the Laboratory Animal Center, Chinese Academy of Sciences. All mice were housed in a temperature‐controlled room (24 ± 2°C) on a 12 h light/12 h dark cycle (lights on at 7:00 a.m.). The mice were housed in groups of four to five mice per cage and maintained under standard laboratory conditions. Mice were allowed free access to food and water in their cages throughout the experiments.

Complete Freund's adjuvant (CFA) (10 μL, Sigma‐Aldrich) was injected into the plantar surface of the left hind paws of the mice using a microliter syringe (Sinopharm Chemical Reagent Co.) to induce persistent inflammatory pain. The persistence of inflammatory pain was confirmed by a second CFA injection (10 μL) on the fourth day. Saline (0.9% sodium chloride solution) was used as the control. The pain threshold was measured using the von Frey test.^16^ All animal protocols were approved by the Animal Care and Use Committee of the University of Science and Technology, China (IACUC 2020‐08‐LJG‐50). This in vivo experimental system in mice replicates aspects of the human pain pathway.^17^

### 2.3 Von Frey test

The baseline thresholds were tested 1 day before CFA injection. After CFA injection, paw withdrawal thresholds were performed once a day.

After an acclimation period of 30 min, we stimulated the plantar surface of the left hindpaw vertically with a series of von Frey filaments (Stoelting) with logarithmically increasing stiffness. The filament was bent for 5 s to the central plantar surface with a sufficient force, and brisk withdrawal or paw flinching was considered as a positive response. The test of withdrawal threshold was repeated five times in each mouse, and if the mouse showed a positive response for three of five tests, the stiffness was judged to be valid and the next level of stiffness was tested until the mouse did not show a positive response to the new stiffness.

### 2.4 Locomotor activity

The mice were placed in locomotor chambers equipped with infrared video recorders, and their activity was monitored for 10 min. The moving distance was analyzed using the DigBehav Animal Behavior Analysis System (Shanghai Jiliang Software Technology).

### 2.5 Elevated plus maze test

The elevated plus maze (EPM) apparatus (65 × 65 cm) was made of black plastic and consisted of two closed and two open arms.^18^ Each arm measured 5 × 30 cm (width × length), and the intersection of the arms measured 5 × 5 cm. The apparatus was elevated 0.5 m above the floor. The experiments were performed between 10:00 h and 16:00 h under light (15 l×). The mice were placed in the center facing the open arm and allowed to move for 5 min. Animal behavior was recorded, and the time spent in the open arms was analyzed. The maze was cleaned with 70% ethanol and distilled water between trials.

### 2.6 Light–dark transition test

The light–dark transition test was performed according to the protocol described in a previous study.^18^ The chamber used for this test (45 × 27 × 30 cm) was divided into two compartments: one part makes up a third of the box (red light: 4 l×) and the other make up two‐thirds for the light (300 l×). Mice were placed in the dark compartment and allowed to freely explore the chamber for 5 min. An infrared video recorder was used to record the movement of the mice. The duration of time spent in the light and dark compartments and the number of mice crossed were assessed.

### 2.7 Marble burying test

The marble burying test was performed in a 29 × 18 × 15 cm cage with husk bedding material distributed in an even layer which was 5 cm deep.^19^ Each mouse was allowed to explore the cage for 10 min without marbles. Then, 20 glass marbles were evenly spaced in a 4 × 5 grid. Each mouse was recorded for 30 min, and the glass marbles buried in the bedding (to 2/3 of their depth) were counted.

### 2.8 Cannula implantation and microinjection

The mice were anesthetized with sodium pentobarbital intraperitonially (70 mg/kg) under aseptic conditions. Next, they were placed in a stereotaxic instrument with non‐puncture ear bars (RWD Life Science), and a 26‐gauge guide cannula was bilaterally implanted in the amygdala (anteroposterior: −1.0 mm; mediolateral: ± 2.8 mm; dorsoventral: −4.7 mm) or in the lateral ventricle (anteroposterior: −1.0 mm; mediolateral: ±3.1 mm; dorsoventral: −5.0 mm). The cannula was anchored to the skull using stainless‐steel screws and dental cement. Bilateral microinfusions were made through 33‐gauge internal cannulae (Plastics One) that extended 1 mm beyond the tips of the guide cannulae to prevent blockage, which were connected to a 10 μL microsyringe mounted on a microinfusion pump (Harvard Apparatus) at an infusion rate of 200 nL/min. For local infusion into a brain region (lateral ventricle and amygdala) and behavioral tests, water‐soluble drugs were dissolved in saline as follows: oxytocin (10 μg/μL) and oxytocin receptor antagonists (10 μg/μL). After injection, an additional 2 min allowed for drug diffusion before concluding the microinfusion process. Oxytocin and oxytocin receptor antagonists were administrated 30 min before each behavior tests.

### 2.9 Virus injection

We purchased rAAV‐oxytocin‐Cre‐WPREs, rAAV‐EF1α‐DIO‐hChR2‐mCherry‐WPREs, rAAV‐EF1α‐DIO‐mCherry‐WPREs, rAAV‐EF1α‐DIO‐hM4D (Gi)‐mCherry‐WPREs‐hGHpA, rAAV‐EF1α‐DIO‐HM4D(Gq)‐mCherry‐hGHpA, rAAV‐OT‐EGFP‐WPREs, Retro‐OT‐Cre‐WPREs, and rAAV‐OT‐GCaMP6s‐WPREs (>5.00 × 1012 TU/mL) from BrainVTA, China. The viruses were bilaterally infused into the PVN of each mouse. The mice were allowed to recover for 4 to 5 weeks before behavioral testing.

### 2.10 Chemogenetic and optogenetic manipulations

To chemogenetic activation PVN neurons, clozapine‐N‐oxide (CNO) (3.0 mg/kg) (Sigma‐Aldrich) was administered intraperitonially, either 30 min before behavioral testing or before killing the animal for brain slicing.

To activate oxytocinergic terminals in the amygdala, optogenetic parameters (ChR2: 10 ms, 30 Hz, 8 s on and 2 s off‐cycle, 15–20 mWatts) were modified as in previous studies with a blue light laser (473 nm).^20^ In the elevated plus maze test and light–dark transition test, the mouse adopted 5 min photostimulation in the homecage before the test in the homecage. Behavioral tests then began without interrupted light. In these two behavioral tests, the mice received 10 min of photostimulation totally each time. Photostimulation was applied simultaneously at the beginning of the bead burying test, and the mice received half an hour stimulation in this test.

### 2.11 Fiber photometry recording

Mice were injected with rAAV‐OT‐GCaMP6s‐WPREs (AAV2/9, >5.00 × 1012 TU/mL, 400 nL) in the PVN. Two optical fibers (diameter of 650 μm, Inper, China) were implanted into the PVN and amygdala. The mice were then allowed to recover for 4–5 weeks before behavioral testing. Optical fiber‐based calcium ion (Ca2+) recordings were performed using a two‐color multichannel optical fiber recording system (410 and 470 nm) (RWD Life Science). The light intensity at the tip of the fiber was 0.03 mW (470 nm) and 0.015 mW (410 nm). Neuronal Ca2+ signals and behavioral videos were recorded simultaneously. The data obtained were analyzed using the analysis software developed by RWD.

### 2.12 Immunofluorescence

Coronal sections (30 μm in thickness) were cut, and brain slices were selected for immunofluorescence staining. Briefly, the brain samples were incubated overnight at 4°C with c‐Fos, oxytocin, and NeuN antibodies. The sections were then incubated with the corresponding secondary antibody (1:500, Invitrogen) at room temperature (25°C) for 2 h followed by visualization using a TI2‐E + A1 R (Nikon, Japan).

### 2.13 Histology

After the behavioral tests, the mice were deeply anesthetized (0.05 mg/10 g Zoletil 50) and perfused with 4% paraformaldehyde. The brains were then removed and kept in a 30% sucrose solution for dehydration for 3 days. For Nissl staining, coronal sections (50 μm thick) were cut on a cryostat (Leica), and Nissl staining was applied to detect the injection sites, followed by visualization using a microscope (Olympus). The data of the animals were kept for analysis only when the cannulae were placed at the correct sites.

### 2.14 Data and statistical analysis

GraphPad Prism 6 was used for data analysis. All data are expressed as mean ± standard error of the mean. Statistical analyses were performed using Student's *t*‐test or one‐ or two‐way analysis of variance with independent or repeated measures by the experimental design. The statistical details of experiments can be found in the figure legends. Statistical significance was defined as *p* < 0.05.

### 2.15 Study design

We established CFA pain‐induced anxiety model in C57BL/6 mice. Pharmacological was used to investigate the role of oxytocin in anxiety‐like behaviors. We used chemogenetic and fiber recordings to modulate and record the activity of the oxytocinergic neurons in the PVN. We then used optogenetic and chemogenetic manipulations to elucidate the role of PVN‐CeA oxytocin projections in anxiety‐like behaviors. All these experiments were designed to demonstrate the modulation of oxytocin as well as PVN‐CeA projection on anxiety behaviors.

## 3 RESULTS

### 3.1 Decreased excitability of PVN OTergic neurons in anxiety mice model

**FIGURE 1:**
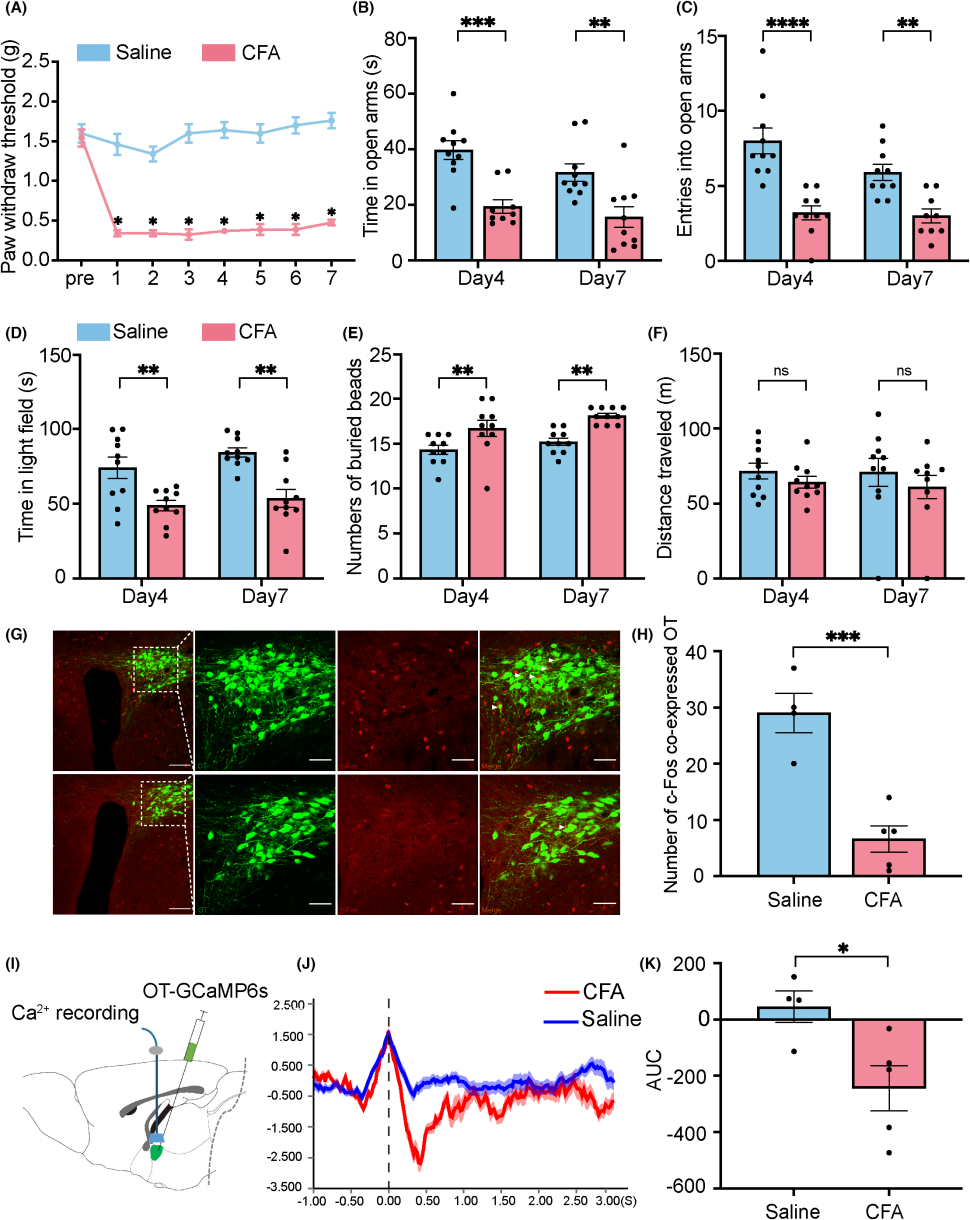
Decreased activity of OTergic neurons in PVN relates to the anxiety behaviors in mice. (A) CFA injection into the left hind paw caused a significant decrease in mechanical paw withdrawal threshold in mice (*n* = 8–10). (B, C) Elevated plus maze test results 4, and 7 days after CFA injection. (D, E) Light–dark transition test and marble burying test results 4, and 7 days after CFA injection. (F) Locomotor activity 4, and 7 days after CFA injection. (G) Overlap of oxytocin marked by OT‐EGFP (green), c‐Fos immunoreactivity (red) in the saline group (up) and CFA group (down). Scale bar of three pictures on the left: 100 μm. Scale bar of picture on the right: 50 μm. (H) Number of c‐Fos and OT‐EGFP co‐localizations (*n* = 4–5). (I) OT‐GcaMP6s microinjection schematic diagram. (J, K) The mean shows that calcium ion (Ca2+) signals decreased in CFA‐injected mice compared to saline‐treated mice when subjected to elevated plus maze test. (K) indicates the area under the curve. The colored bars indicate ΔF/F (%) (*n* = 5). CFA, Complete Freund's adjuvant.

Pain, as one common clinical symptom of diverse disease, is also a major cause for anxiety. Here, we developed anxiety mice model by CFA‐induced inflammatory pain. Briefly, mice were injected with CFA (Figure 1A). EPM, light–dark transition test and beads burying tests were performed at day 4 and 7 to assess the anxiety behaviors. The results showed that mice with inflammatory pain displayed less activity in the open arm in EMP test (Day 4—saline: 39.78 ± 10.55 s, Day 4—CFA: 19.51 ± 7.32 s, *n* = 10, *p* = 0.0007; Day 7—saline: 31.73 ± 10.05 s, Day 7—CFA: 15.69 ± 11.84 s, *n* = 10, *p* = 0.0070; Figure 1B; Day 4—saline: 8 ± 2.569, Day 4—CFA: 3.2 ± 1.400, *n* = 10, *p* < 0.0001; and Day 7—saline: 5.9 ± 1.578, Day 7—CFA: 3 ± 1.333, *n* = 10, *p* = 0.0022; Figure 1C), spent less time in the light field in the light–dark transition test (Day 4—saline:73.921 ± 21.598 s, Day 4—CFA: 48.77 ± 10.495 s, *n* = 10, *p* = 0.0085; and Day 7—saline: 84.248 ± 9.357 s, Day 7—CFA: 53.644 ± 18.130, *n* = 10, *p* = 0.0018; Figure 1D) and buried more marbles in marble burying test (Day 4—saline:14.3 ± 1.552, Day 4—CFA: 16.7 ± 2.722, *n* = 10, *p* = 0.0068; and Day 7—saline: 15.2 ± 1.249, Day 7—CFA: 18.1 ± 0.831, *n* = 10, *p* = 0.0014; Figure 1E) as compared to mice without inflammatory pain, suggesting a sustainable anxiety phenotype by CFA‐induced inflammatory pain. CFA‐induced pain did not change the general locomotor ability of the mice (Day 4—saline: 71.660 ± 15.912 m, Day 4—CFA: 64.144 ± 11.784 m, *n* = 10, *p* = 0.7343; and Day 7—saline: 70.889 ± 28.034 m, Day 7—CFA: 61.108 ± 23.205 m, *n* = 10, *p* = 0.5971; Figure 1F).

OT neurons in PVN regulate social brewards, and its aberrant activity is involved in the pathological process of mental disorders.^21^ The detection of a neuronal activation marker c‐Fos showed that the c‐Fos expressing neurons, as well as the percentage of c‐Fos/oxytocin double‐labeled neurons in the PVN, drastically decreased in the CFA anxiety model group (Figure 1G,H). Next, we further performed fiber photometry test to analysis the excitability of OTergic neuron in PVN. We injected rAAV‐OT‐ GCaMP6s into the PVN and inserted the optic fiber to record the activity of oxytocin neurons in the PVN via fiber photometry (Figure 1I). The results showed that the Ca^2+^ signals were lower in the anxiety model group during the EPM test, suggesting a reduced activity of oxytocin neurons in the PVN in anxiety model (Figure 1J,K). These results indicate a potential involvement of OTergic system in the pathological process of anxiety.

### 3.2 Oxytocin perform anti‐anxiety effect

**FIGURE 2:**
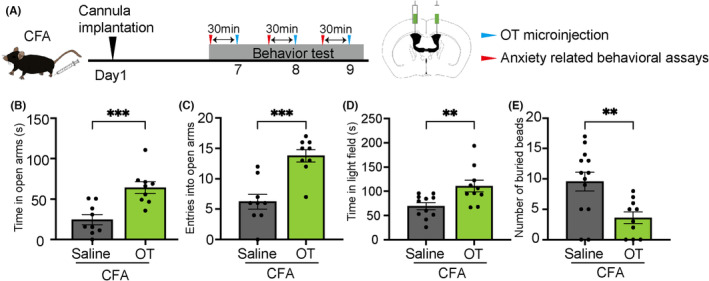
Microinjections of oxytocin into the lateral ventricle prevent anxiety‐like behavior. (A) Schematic diagram and timeline of the experiment. (B–E) Elevated plus maze, light–dark transition, and marble burying tests, respectively, of CFA‐injected mice after 20 μg bilateral oxytocin microinjection. Error bars indicate standard error. *p < 0.05, **p < 0.01, ***p < 0.001, ****p < 0.0001 compared with saline‐treated control group, two‐way ANOVA with Bonferroni's post hoc test. CFA, Complete Freund's adjuvant; PVN, paraventricular nucleus; ANOVA, analysis of variance; N.S, not significant.

To further verify the critical role of OTergic neurons in the PVN for anxiety behaviors, we supplied oxytocin to the brain by lateral ventricle infusion (Figure 2A). The results showed that OT administration rescued the CFA‐induced inflammatory pain‐induced anxiety behaviors. The results of the EPM test showed that mice with microinjection of oxytocin into the lateral ventricle spent more time in the open arms (saline: 24.64 ± 6.228 s, OT: 64.18 ± 7.203 s, *n* = 9, *t* = 4.152, *p* = 0.0007; Figure 2B) and displayed more open arm entries (saline: 6.222 ± 1.211, OT: 13.78 ± 1.011, *n* = 9, *t* = 4.790, *p* = 0.0002; Figure 2B). Moreover, the results of the light–dark transition test showed that decreased time in the light field by the pain from CFA was blocked by the microinjection of oxytocin (saline: 69.33 ± 7.189 s, OT: 110.7 ± 12.22 s, *n* = 10–11, *t* = 2.984, *p* = 0.0076; Figure 2D), and marble burying test showed that mice with oxytocin administration buried less marbles (saline: 9.538 ± 1.538, OT: 3.600 ± 0.9568, *n* = 10–13, *t* = 3.044, *p* = 0.0062; Figure 2E). Microinjection of OT into naïve mice did not affect the anxiety‐like performance (Supplementary Figure S1) and the cognitive function measured by open field test and novel object recognition test (Supplementary Figure S2).

### 3.3 Activation of PVN oxytocin neurons blocks anxiety‐like behaviors following inflammatory pain

**FIGURE 3:**
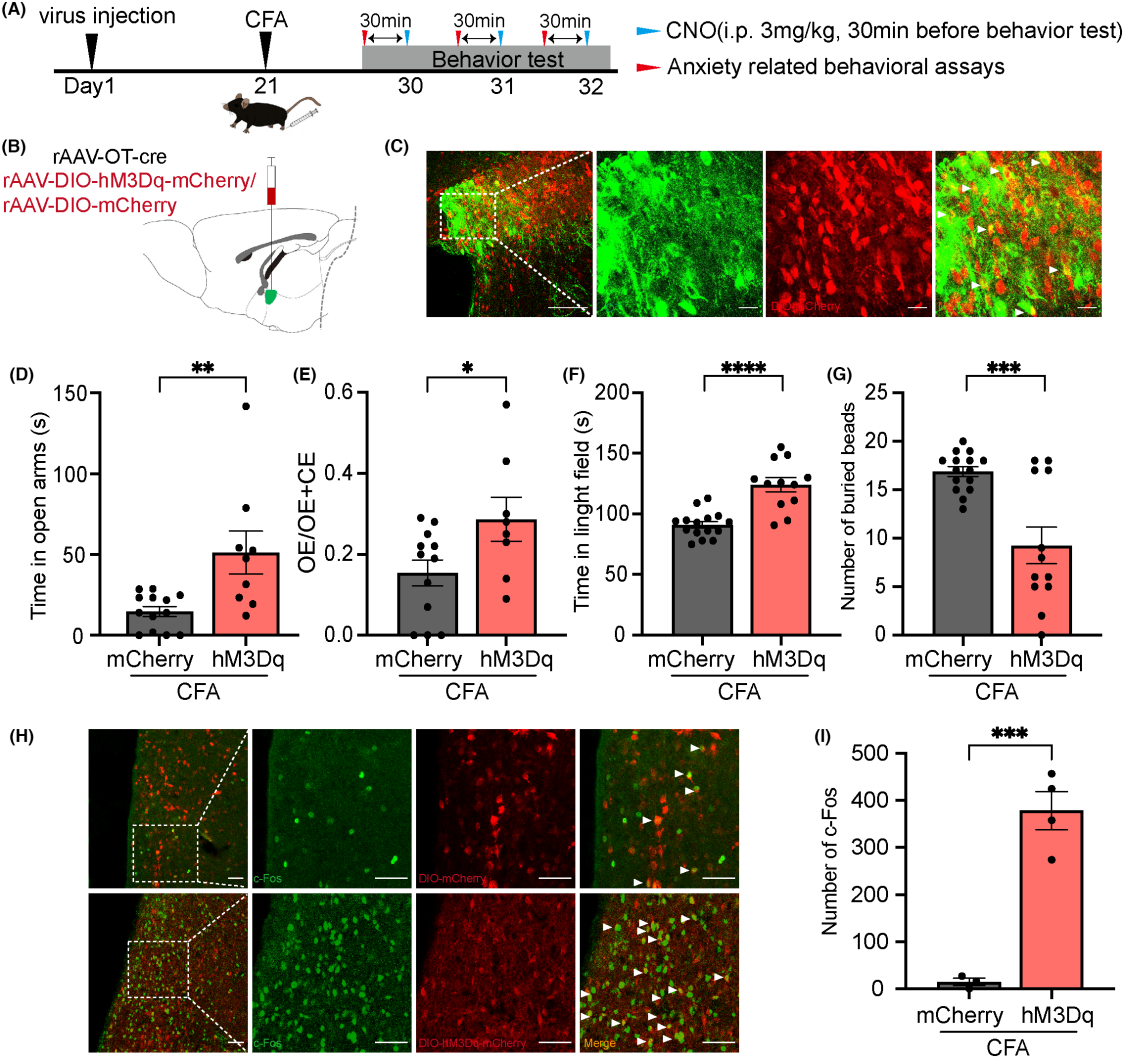
Activation of PVN oxytocin neurons blocked pain‐induced anxiety‐like behaviors. (A) Timeline of the experiment. (B) Schematic diagram of rAAV‐oxytocin‐Cre‐WPREs and rAAV‐EF1α‐DIO‐hM3Dq‐mCherry‐hGHpA microinjections. (C) Co‐localization of immunoreactive DIO‐ hM3Dq ‐mCherry (red) and oxytocin (green). Scale bar of three pictures on the left: 100 μm. Scale bar of picture on the right: 50 μm. (D–G) Results of the elevated plus maze, light–dark transition, and marble burying tests after clozapine‐N‐oxide (CNO) intraperitoneal injection 30 min prior. (H) Representative images of the PVN illustrating c‐Fos in neurons expressing DIO‐mCherry group(up) and DIO‐hM3Dq‐mCherry group(down). Scale bar of three pictures on the left: 100 μm. Scale bar of picture on the right: 50 μm. (I) Number of c‐Fos and mCherry co‐localizations. OE/OE + CE means ratios of entries into open arms to the sum of entries into open arms and entries into closed arms. *p < 0.05, **p < 0.01, ***p < 0.001, ****p < 0.0001 compared with mCherry group in unpaired two‐tailed *t*‐test. CNO, clozapine‐N‐oxide. Error bars indicate standard error.

To verify the crucial role of PVN oxytocin neurons in pain‐related anxiety‐like behaviors, we used a Cre‐recombinase‐dependent viral vector approach to selectively express Gq‐coupled DREADDs (designer receptors exclusively activated by designer drugs) in oxytocin neurons in the PVN. The detailed timeline of the experiments is shown in Figure 3A. The OT‐Cre and DIO‐hM3Dq‐mCherry virus was injected into the PVN (Figure 3B,C). Four weeks after infusion of the virus, these mice underwent the same CFA injection procedure. The results showed that chemogenetic activation of the oxytocin neurons in the PVN by CNO inflammatory pain‐induced anxiety‐like behaviors as measured by the EPM, light–dark transition, and marble burying tests (Figure 3D: mCherry:14.77 ± 3.114 s, hM3Dq: 51.24 ± 13.26 s, *t* = 3.158, *p* = 0.0049; Figure 3E: mCherry:0.1542 ± 0.03192, hM3Dq:0.2863 ± 0.05441, *t* = 2.240, *p* = 0.0379; Figure 3F: mCherry: 91.01 ± 2.779 s, hM3Dq:124.1 ± 5.876 s, *t* = 5.432, *p* < 0.0001; and Figure 3G: mCherry: 16.87 ± 0.5152, hM3Dq:9.250 ± 1.887, *t* = 4.287, *p* = 0.0002; *n* = 9–13). The effectiveness of hM3Dq was measured by the expression of c‐Fos (Figure 3H,I).

### 3.4 Inhibition of PVN oxytocin neurons induces anxiety

**FIGURE 4:**
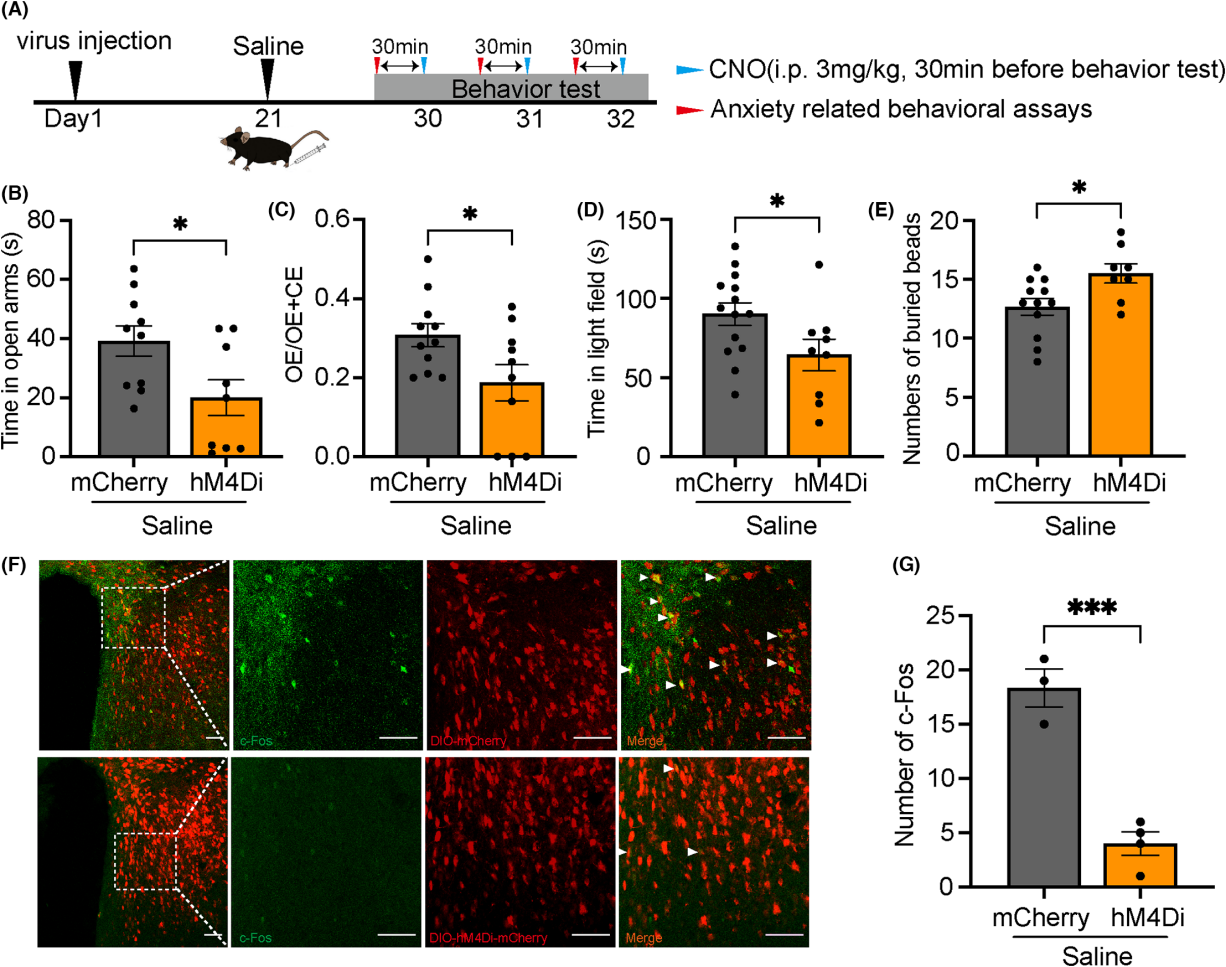
Inhibition of the PVN oxytocin neurons increased pain‐induced anxiety‐like behaviors. (A) Timeline of the experiment. (B–E) Elevated plus maze, light–dark transition, and marble burying tests after clozapine‐N‐oxide (CNO) intraperitoneal injection for 30 min. (F) Representative images of the PVN illustrating c‐Fos in neurons expressing DIO‐mCherry and DIO‐hM3D(Gi)‐mCherry. Scale bar of three pictures on the left: 100 μm. Scale bar of picture on the right: 50 μm. (G) Number of c‐Fos. OE/OE + CE means ratios of entries into open arms to the sum of entries into open arms and entries into closed arms. *p < 0.05, ***p < 0.001 compared with mCherry group in unpaired two‐tailed *t*‐test. Error bars indicate standard error.

To clarify the role of PVN oxytocin neurons in the initiation of anxiety‐like behaviors, we infused rAAV‐oxytocin‐Cre and rAAV‐EF1α‐DIO‐hM4Di‐mCherry into both sides of PVN. We investigated whether inhibition of PVN oxytocin neurons affected anxiety‐like behaviors in mice. The timeline of the experiments is shown in Figure 4A. Mice with inhibited PVN oxytocin neurons spent significantly less time in the open arms (mCherry: 39.19 ± 5.178 s, hM4Di:19.99 ± 6.025 s, *n* = 9–10, *t* = 2.430, *p* = 0.0265; Figure 4B) and entrie open arms fewer times (mCherry: 0.3073 ± 0.02905, hM4Di: 0.1870 ± 0.04612, *n* = 10–11, *t* = 2.251, *p* = 0.0364; Figure 4C). In the light–dark transition test, mice also spent less time in the light field (mCherry: 90.18 ± 7.138 s, hM4Di:64.39 ± 10.03 s, *n* = 10–14, *t* = 2.153, *p* = 0.0431; Figure 4D) as compared to mice without inhibited PVN oxytocin neurons. Additionally, the number of buried marbles increased (mCherry:12.67 ± 0.7213, hM4Di:15.50 ± 0.8238, *n* = 8–12, *t* = 2.550, *p* = 0.0201; Figure 4E). The injection of CNO led to a decrease in c‐Fos expression in the PVN (Figure 4F,G).

### 3.5 CeA is involved in the anti‐anxiety effects of oxytocin

**FIGURE 5:**
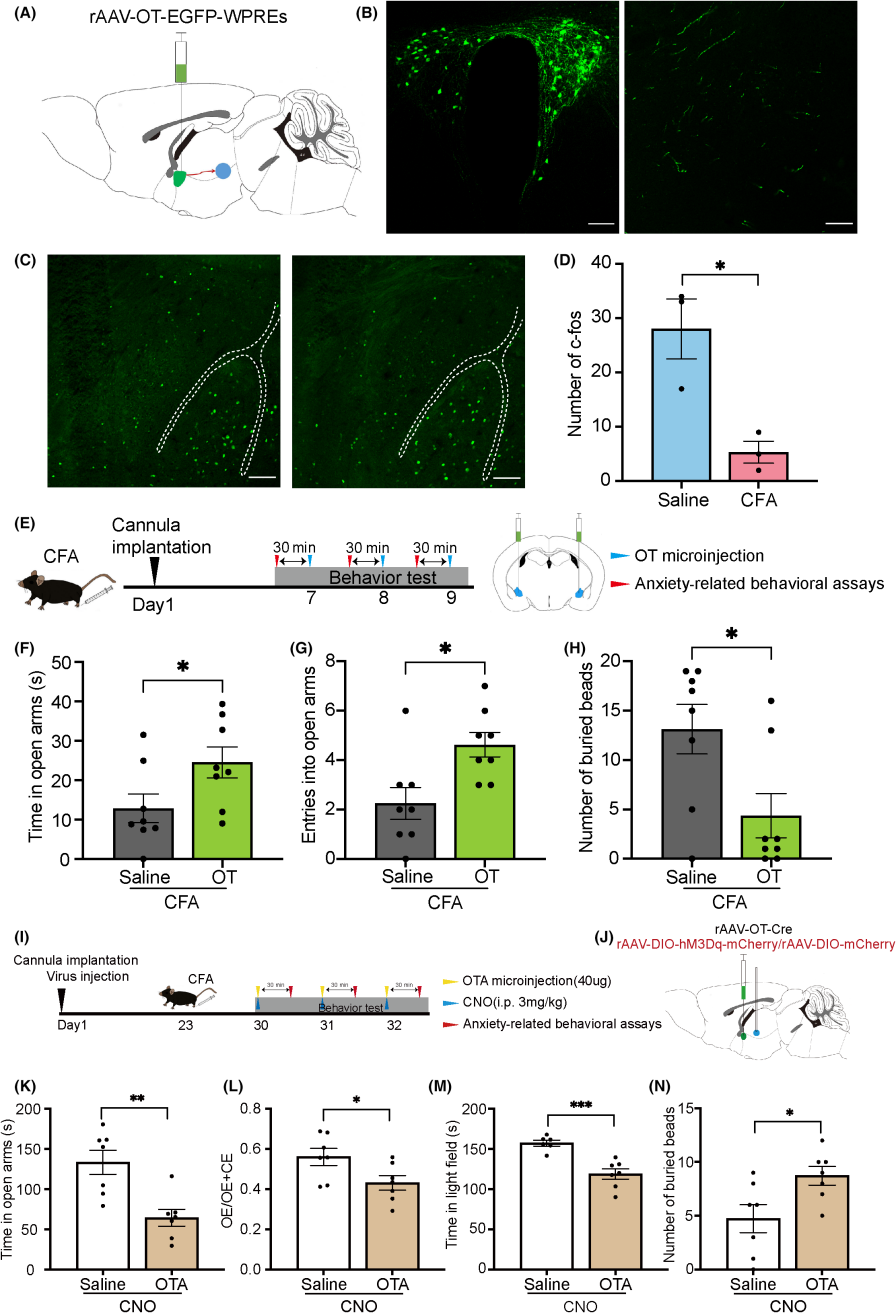
Oxytocin bilateral microinjection in the CeA blocks inflammatory pain‐induced anxiety‐like behaviors. (A) OT‐EGFP microinjection schematic diagram. (B) Immunoreactive OT‐EGFP (green). Left: OT‐EGFP in the paraventricular nucleus (PVN); right: projection fiber in the central nucleus of the amygdala (CeA). Scale bar: 100 μm. (C) Immunoreactive c‐Fos (green) in the complete Freund's adjuvant (CFA) group compared with the saline group. Scale bar: 100 μm. (D) Number of c‐Fos in the CeA. (E) Timeline of the experiment and oxytocin microinjection schematic diagram. (F–H) Results of elevated plus maze and marble burying tests of CFA‐injected mice after 20 μg oxytocin microinjection into the CeA. (I) Timeline of the experiment. (J) Schematic diagram of rAAV‐oxytocin‐Cre‐WPREs and rAAV‐EF1α‐DIO‐hM3Dq‐mCherry‐hGHpA microinjections with cannula implantation. (K–N) Elevated plus maze, light‐dark transition, and marble burying tests after clozapine‐N‐oxide (CNO) intraperitoneal injection and OTA (40 ug for each mouse) microinjection for 30 min. *p < 0.05, **p < 0.01 compared with saline group in unpaired two‐tailed *t*‐test. Error bars indicate standard error.

The amygdala is a brain region associated with emotions and has a large number of oxytocin receptors.^22^ We investigated whether the CFA‐induced decrease in PVN oxytocin neuron activity led to decreased oxytocin in the amygdala. We first injected tracing virus OT‐EGFP‐WPREs into the PVN and found significant projecting fibers in the CeA (Figure 5A,B). To clarify whether CeA is involved in anxiety behaviors, we examined the expression of c‐Fos after CFA injection and found reduced c‐Fos expression in the CeA (Figure 5C,D). We hypothesized that the decreased OTergic transmission in the CeA might mediate the process of anxiety behavior. Further results were compatible with this assumption, and showed that OT microinfusion into CeA rescued the inflammatory pain‐induced anxiety‐like behaviors (Figure 5E), which were assessed by EMP and marble burying tests (saline: 12.86 ± 3.642 s, OT: 24.52 ± 3.907 s, *n* = 7–8, *t* = 2.183, *p* = 0.0465; Figure 5F; saline: 2.250 ± 0.6478, OT: 4.625 ± 0.4978, *n* = 7–8, *t* = 2.907, *p* = 0.0115; Figure 5G; saline:13.13 ± 2.503, OT: 4.375 ± 2.244, *n* = 8, *t* = 2.603, *p* = 0.0209; Figure 5H).

Oxytocin receptors are intensively expressed in the CeA. To clarify if oxytocin receptor mediates anti‐anxiety effect in activating PVN oxytocin neurons, we injected rAAV‐Oxytocin‐Cre and DIO‐hM3Dq‐mCherry into both sides of PVN while implanted the cannula into CeA. Oxytocin receptor antagonists (OTAs), L‐368,899 hydrochloride were microinjected into CeA to access if antagonism oxytocin receptor could block the behavioral effects of activation PVN oxytocin neurons. The timeline and schematic of the experiments is shown in Figure 5I,J. Mice with OTA microinjection spent less time in the open arms (Saline: 133.3 ± 14.96 s, OTA:64.30 ± 10.50s, *n* = 7, *t* = 3.775, *p* = 0.0026; Figure 5K) and entries open arms fewer times (Saline: 0.5603 ± 0.04234, OTA:0.4309 ± 0.03585, *n* = 7, *t* = 2.333, *p* = 0.0379; Figure 5L). In the light–dark transition test, mice also spent less time in the light field (Saline: 157.2 ± 3.704 s, OTA:118.7 ± 6.474 s, *n* = 7, *t* = 4.923, *p* = 0.0005; Figure 5M) as compared to mice without block of oxytocin receptors. Also, the number of buried marbles increased (Saline: 4.714 ± 1.304, OTA:8.714 ± 0.8650, *n* = 7, *t* = 2.556, *p* = 0.0252; Figure 5N). The result revealed that PVN‐CeA oxytocinergic circuit performed anti‐anxiety effect via oxytocin receptors.

### 3.6 Activation of the PVN‐CeA oxytocinergic projection prevents anxiety‐like behaviors

**FIGURE 6:**
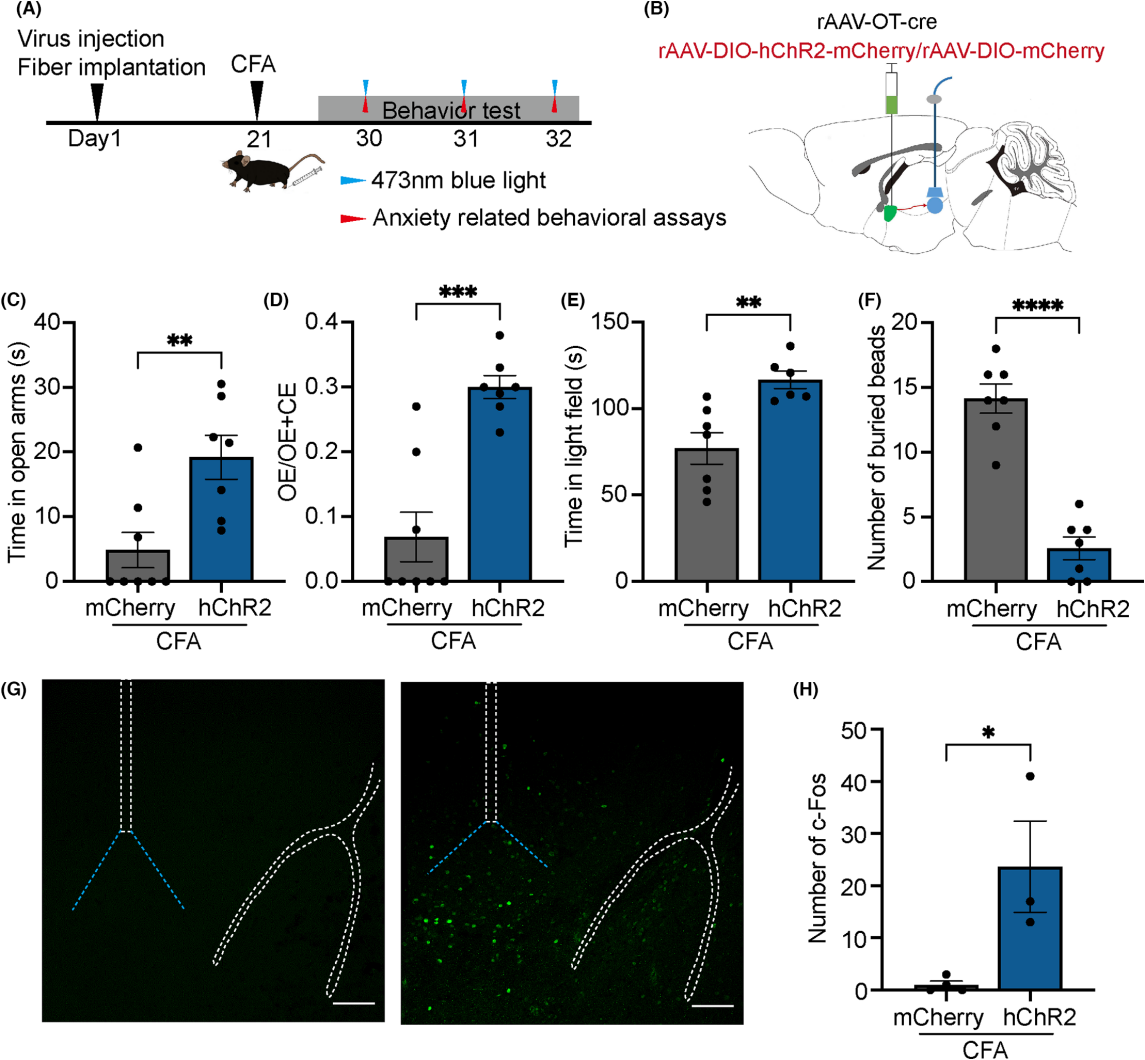
Activation of PVN‐CeA oxytocin neuron projection blocked inflammatory pain‐induced anxiety‐like behaviors. (A) Left: timeline of the experiment; Right: schematic representing the OT‐Cre and DIO‐ChR2‐mCherry microinjection sites, and fiber implant location. (B) Schematic diagram of rAAV‐oxytocin‐Cre‐WPREs, rAAV‐EF1α‐DIO‐ hChR2 ‐mCherry‐hGHpA microinjections and fiber implant. (C–F) Results of elevated plus maze, light–dark transition (stimulation parameters: 20 Hz, 10–20 mW, 10 ms, 3 s on 2 s off, 5 min), marble burying (20 Hz, 5 mW, 5 ms, 3 min on, 2 min off, 30 min) with 473 nm blue light. (G) Representative images of the CeA illustrating c‐Fos in neurons expressing DIO‐hChR2‐mCherry projection fibers. Scale bar: 100 μm. (H) Number of c‐Fos. *p < 0.05, **p < 0.01, ***p < 0.001, ****p < 0.0001 compared with mCherry group in unpaired two‐tailed *t*‐test. Error bars indicate standard error.

To further explore whether activating PVN oxytocin neuron terminals in the CeA decreases inflammatory pain‐induced anxiety‐like behavior, we injected a combination of rAAV‐oxytocin‐Cre with rAAV‐Ef1α‐DIO‐ChR2‐mCherry or rAAV‐Ef1α‐DIO‐mCherry into the right PVN. We implanted an optic fiber into the right CeA, reliably responding to pulses of 473 nm light (Figure 6A,B). Mice with optical activation of CFA groups expressing ChR2‐mCherry played for an increased time in the open arms (mCherry: 4.871 ± 2.724 s, hChR2:19.18 ± 3.383 s, *n* = 8, *t* = 3.330, *p* = 0.0054; Figure 6C), had greater entries in the open arms (mCherry: 0.06875 ± 0.03815, hChR2:0.3000 ± 0.01773, *n* = 8, *t* = 5.235, *p* = 0.0002; Figure 6D) were greater, spent increased time in the light field of the light–dark transition test (mCherry:76.99 ± 9.081 s, hChR2:116.7 ± 5.058 s, *n* = 6–7, *t* = 3.639, *p* = 0.0039; Figure 6E) and buried less marbles (mCherry: 14.14 ± 1.122, hChR2: 2.571 ± 0.8690, *n* = 7, *t* = 8.155, *p* < 0.0001; Figure 6F) as compared to mice without optical activation. These results show that optical activation of the PVN‐CeA can reduce inflammatory pain‐induced anxiety‐like behaviors in mice. 473 nm blue light increased c‐Fos expression in the CeA (Figure 6G,H).

### 3.7 Inhibition of the PVN‐CeA oxytocin circuit prevents anxiety‐like behaviors following inflammatory pain

**FIGURE 7:**
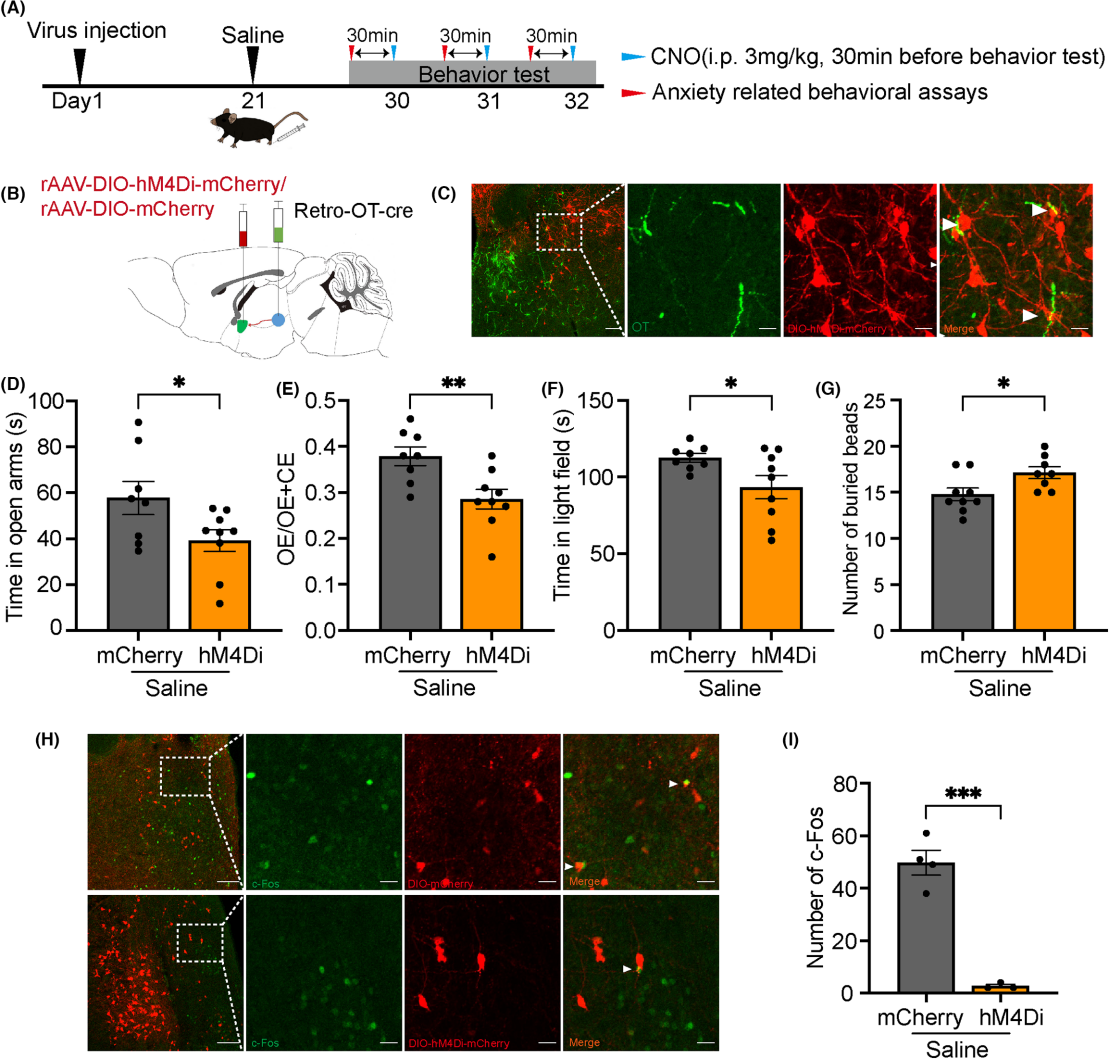
Inhibition of the PVN‐CeA oxytocin projection increased pain‐induced anxiety‐like behaviors and this projection performed anti‐anxiety effect by oxytocin receptor. (A) Timeline of the experiment. (B) Schematic diagram of Retro‐oxytocin‐Cre‐WPREs and rAAV‐EF1α‐DIO‐hM4Di‐mCherry‐hGHpA microinjections. (C) Co‐localization of immunoreactive DIO‐hM4Di‐mCherry (red) and oxytocin (green). Scale bar of three pictures on the left: 100 μm. Scale bar of picture on the right: 50 μm. (D–G) Elevated plus maze, light–dark transition, and marble burying tests after clozapine‐N‐oxide (CNO) intraperitoneal injection for 30 min. (H) Co‐localization of immunoreactive DIO‐ hM4Di‐mCherry (red) and c‐Fos (green) in the paraventricular nucleus (PVN). Scale bar of three pictures on the left: 100 μm. Scale bar of picture on the right: 50 μm. (I) Number of c‐Fos. OE/OE + CE means ratios of entries into open arms to the sum of entries into open arms and entries into closed arms. *p < 0.05, **p < 0.01, ***p < 0.001 compared with mCherry group in unpaired two‐tailed *t*‐test. Error bars indicate standard error.

To further clarify the function of the PVN‐CeA oxytocin projection, we injected Retro‐Oxytocin‐Cre into both sides of CeA and injected rAAV‐Ef1α‐DIO‐hM4Di‐mCherry or rAAV‐Ef1α‐DIO‐mCherry into both sides of PVN. Virally labeled neurons were observed in the PVN, which corroborated the results of retrograde mapping experiments (Figure 7A–C). The inhibition of PVN‐CeA oxytocin projection significantly decreased the time which mice spent in the open arms (mCherry: 57.78 ± 7.244 s, hM4Di: 39.27 ± 4.756 s, *n* = 9–10, *t* = 2.183, *p* = 0.0454; Figure 7D) and the frequency of entries into the open arms (mCherry:0.3788 ± 0.02030, hM4Di: 0.2856 ± 0.02109, *n* = 8–9, *t* = 3.164, *p* = 0.0064; Figure 7E). Mice also spent less time in the light field in the light–dark transition test (mCherry: 112.6 ± 2.774 s, hM4Di:93.45 ± 7.624 s, *n* = 8–9, *t* = 2.247, *p* = 0.0401; Figure 7F). Additionally, the number of buried marbles increased (mCherry: 14.78 ± 0.6827, hM4Di: 17.13 ± 0.6391, *n* = 8–9, *t* = 2.490, *p* = 0.0250; Figure 7G). The injection of CNO led to a decrease in c‐Fos expression in the PVN (Figure 7H,I).

## 4 DISCUSSION

In our study, we found that the anxiety‐like behaviors in mice could be blocked by oxytocin administration. The induction of anxiety‐like behaviors was accompanied by decreased activity of PVN oxytocin neurons. The modulation of oxytocin neurons in the PVN affects anxiety‐like behaviors related to inflammatory pain. We further found that neurons in the CeA were inhibited in CFA‐treated mice and intra‐CeA injection of oxytocin prevented anxiety behaviors. Finally, we found that activation of the PVN‐CeA oxytocin projection prevented anxiety‐like behaviors induced by inflammatory pain. These results reveal the important role of oxytocin projections of the PVN‐CeA circuit in anxiety‐like behaviors.

The role of oxytocin in anxiety remains controversial. Although most studies suggest that oxytocin exerts anxiolytic effects, some studies have proposed that anxiety is a side effect of oxytocin.^23^, ^24^, ^25^ According to our research, the injection of CFA could induce stable anxiety symptoms 4 days later. Our experiment confirmed microinjection of oxytocin could perform anti‐anxiety effect in this anxiety model, which matched to most research.

Oxytocin has been reported to play a key role in the regulation of anxiety induced by paternal deprivation^20^ and performed significant analgesic effect.^26^ We used CFA pain‐anxiety model to evaluate the effect of oxytocin on comorbidity of pain and anxiety. The results of our experiments show that oxytocin treatment significantly blocked anxiety‐like behaviors following inflammatory pain, which is similar to the effect of oxytocin to other anxiety model.

Oxytocin neurons project to different brain regions. Previous study shows that PVN‐CeA projection is critically involved in the regulation of pain‐related mood disorders.^27^ This circuit may also play an important role in comorbidity of pain and anxiety. The terminals of oxytocin neuron fibers can be seen in the amygdala, confirming the existence of PVN‐CeA oxytocin projection. In our experiment, optogenetic and chemogenetic were used to verify the function of PVN‐CeA oxytocinergic projection and the activation of this circuit can alleviate the anxiety‐like behavior in mice.

Oxytocin has two main receptors, oxytocin receptors and vasopressin receptors. Oxytocin receptors are intensively distributed in the amygdala. A major finding of our experiment is that microinjection of OT directly into the CeA could alleviate the change to anxiety‐like behavior. But activation of CeA and its origin brain region plus OTA re‐exhibit the anxiety behavior. This result is consistent with other research which focuses on other brain region.^28^

Our findings suggest that modulating the activity of oxytocin neurons in the PVN may lead to the change in the anxiety‐like performance. We used Cre‐DIO hM4D(Gq) and hM4D(Gi) to confirm this hypothesis by activating or inhibiting the oxytocin neurons in the PVN, respectively. Activating the oxytocin neurons was found to reduce anxiety behaviors, while inhibiting increased the behaviors. These results confirm that oxytocin neurons in the PVN are involved in regulating anxiety behaviors. Previous reports have indicated that oxytocin levels in the hypothalamus may be low in those who suffer from chronic pain. Activation of oxytocin neurons in the PVN can provide relief.^29^ Anxiety is accompanied by a decrease in oxytocin.^30^, ^31^, ^32^ The findings of our study reveal that oxytocin neurons are altered in inflammatory pain‐induced anxiety‐like behaviors and regulate these behaviors in mice.

We used optogenetics to specifically activate the terminals of the PVN‐CeA circuit and found that activating the oxytocinergic terminals significantly minimized anxiety‐like behaviors in mice and could increase activation in downstream brain regions. Therefore, we demonstrated that this circuit could regulate anxiety‐like behaviors and showed its function in regulating emotions.

Previous studies have shown that oxytocin neurons in the PVN are closely related to social behavior and emotional disorders.^13^, ^14^ PVN oxytocin neurons project to different brain regions and modulate different physiological and pathological processes. To illustrate, the PVN‐prelimbic area regulates sociability and anxiety‐like behavior under paternal deprivation. Furthermore, the PVN‐ventral tegmental area is closely related to paternal behavior.^20^ Our study builds upon a previous study by showing that the PVN‐CeA oxytocin projection is critically involved in anxiety disorders related to inflammatory pain.

## 5 CONCLUSIONS

In summary, the findings of the present study revealed that oxytocin plays a key role in inflammatory pain‐induced anxiety‐like behaviors. The results suggest that the reduced activity of PVN oxytocin neurons and its projections to the CeA contribute to anxiety‐like behaviors induced by inflammatory pain. Given that anxiety symptoms are often accompanied by pain in the clinical setting, this work has important clinical implications, as it suggests that oxytocin and the PVN‐CeA oxytocin inputs are promising targets for therapeutic intervention to prevent comorbidity of pain and anxiety.

## AUTHOR CONTRIBUTIONS

Z‐Q L, J‐G L, and Y‐J L designed the experiments. Y‐J L, W‐J D, and R L performed the experiments with the assistance of G‐Y Z, B‐L Y, Z‐H S, and Y‐W Y. Statistical data analysis was performed by Y‐J S and Y‐J L. This manuscript was written by Y‐J L and revised by Z‐Q L, J‐G L. Funding acquisition was done by Z‐Q L and Q L.

## CONFLICT OF INTEREST STATEMENT

The authors declare no conflict of interest.

## Supporting information


Supplementary Figure S1.

Supplementary Figure S2.

## Data Availability

The data used to support the findings of this study are available from the corresponding author upon request.
