# Supplementary figures and images for: Paraventricular nucleus‐central amygdala oxytocinergic projection modulates pain‐related anxiety‐like behaviors in mice
Source: CNS Neurosci Ther. 2023 May 29;29(11):3493–506. doi: 10.1111/cns.14282 (PMC10580334; doi:10.1111/cns.14282)

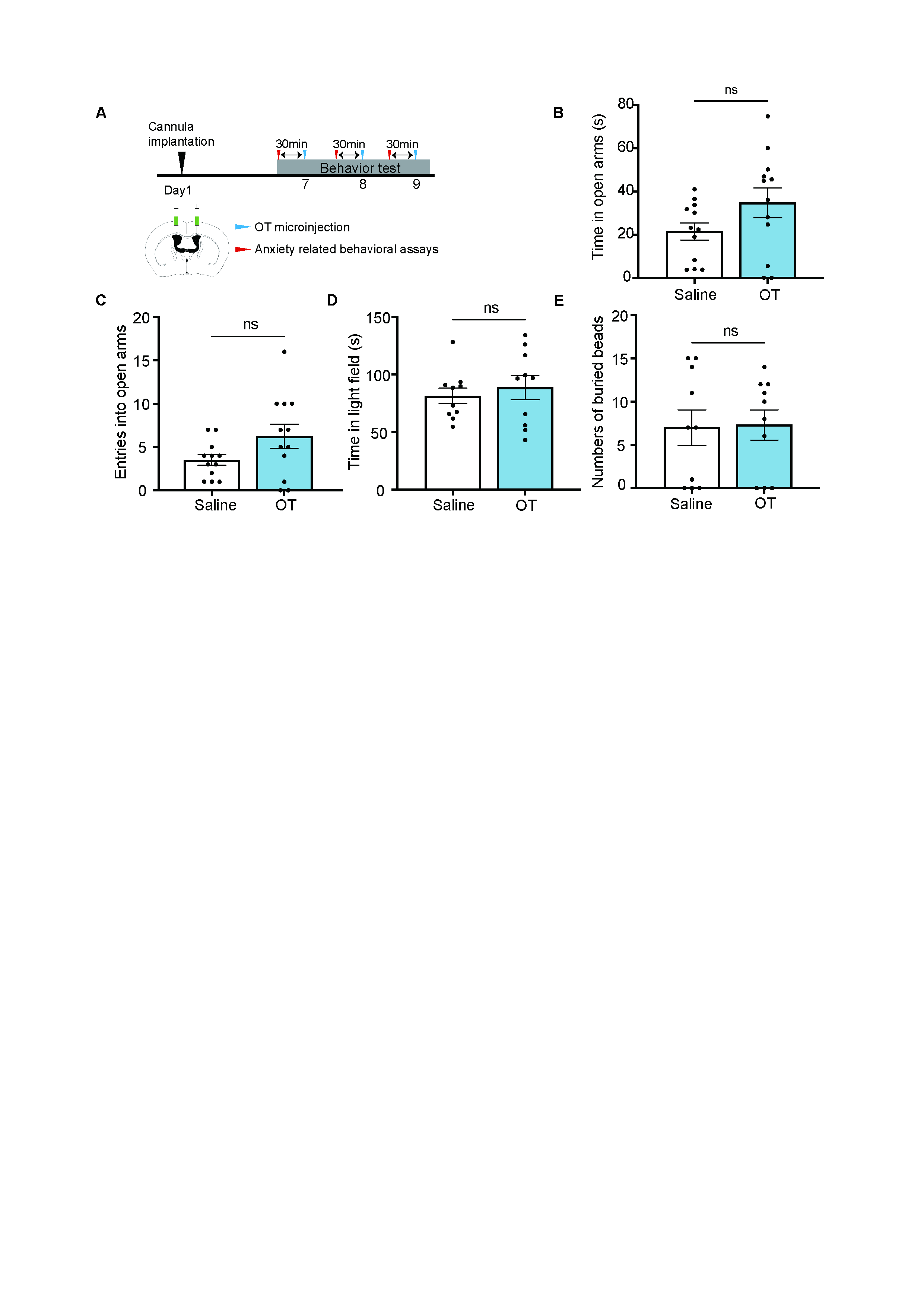

Supplement: Supplementary file 1 — Supplementary Figure S1. [file CNS-29-3493-s002.tif]

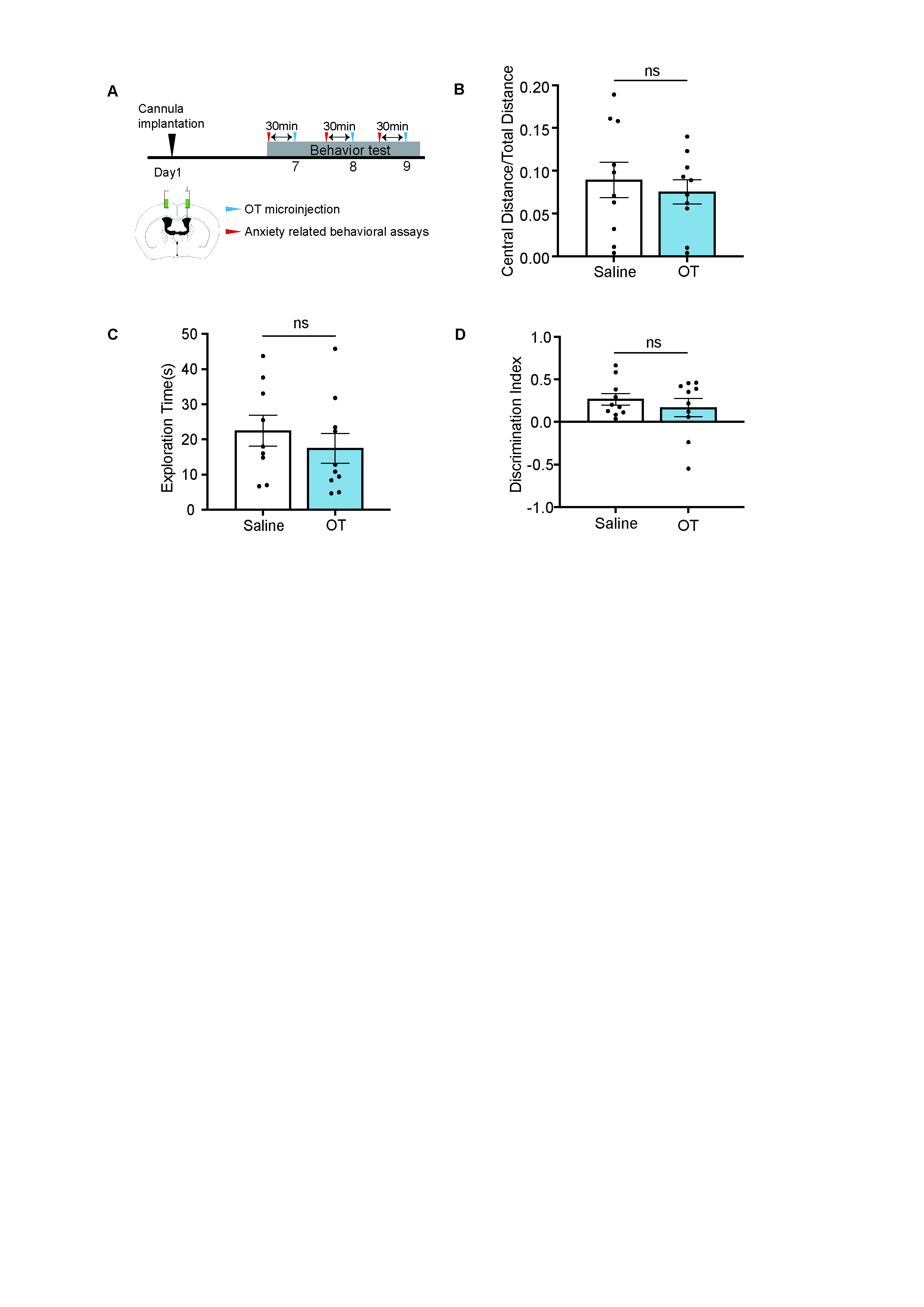

Supplement: Supplementary file 2 — Supplementary Figure S2. [file CNS-29-3493-s001.tif]
